# KSHV LANA upregulates the expression of epidermal growth factor like domain 7 to promote angiogenesis

**DOI:** 10.18632/oncotarget.23456

**Published:** 2017-12-19

**Authors:** Suhani Thakker, Roxanne C. Strahan, Alexandra N. Scurry, Timsy Uppal, Subhash C. Verma

**Affiliations:** ^1^ Department of Microbiology and Immunology, School of Medicine, University of Nevada, Reno, NV 89557, USA

**Keywords:** Kaposi's sarcoma, angiogenesis, cell proliferation

## Abstract

Kaposi’s sarcoma (KS) is a highly-vascularized tumor characterized by inflammation and extensive neo-angiogenesis. The KS tumor microenvironment is rich in inflammatory and pro-angiogenic cytokines. Here, we report that the expression of Epidermal growth factor-like domain 7 (EGFL7) is upregulated in Kaposi’s sarcoma-associated herpes virus (KSHV) infected cells. EGFL7 is a secreted pro-angiogenic cytokine that has been implicated in angiogenesis and the proliferation of endothelial cells during many pathological conditions. Our data show that KS tumors as well as primary effusion lymphoma cells have increased levels of EGFL7 compared to the uninfected cells. We determined that the expression of a KSHV latent protein, LANA (latency-associated nuclear antigen), is the main viral factor responsible for this upregulation. The modulation of EGFL7 expression by LANA involves sequestration of death domain-associated protein 6 (Daxx) from the EGFL7 promoter. Daxx acts as a suppressor of promoter activity by binding to the avian erythroblastosis virus E26 oncogene homolog 1 (Ets-1), which is the core transcription factor required for the expression of EGFL7. We additionally show that the upregulation of EGFL7 by LANA contributes to the promotion of angiogenesis since siRNA-mediated knockdown of EGFL7 reduced *in vitro* tubulogenesis in LANA-expressing HUVEC cells. EGFL7 promotes angiogenesis through autocrine as well as paracrine mechanisms as the supernatant from LANA expressing cells depleted of EGFL7 showed reduced tubulogenesis. This study for the first time demonstrates EGFL7 to be an important angiogenic molecule secreted during KSHV infection that could be exploited for blocking KSHV associated malignancies in conjugation with other anti-angiogenic therapies.

## INTRODUCTION

Kaposi’s sarcoma-associated herpes virus (KSHV) is an oncogenic virus responsible for multiple human malignancies including Kaposi’s sarcoma (KS) and two-lymphoproliferative diseases- primary effusion lymphoma (PEL or BCBL) and multicentric Castleman’s disease [1–3]. KS is the most common cancer associated with HIV infection and remains a leading cause of morbidity and mortality in AIDS patients. It is a highly vascular tumor of endothelial origin that develops due to a complex interplay of immune evasion, inflammation and angiogenesis. Angiogenesis, the process of growing new blood vessels from preexisting ones, is a hallmark of KS tumors. The significance of angiogenesis in KS tumors is highlighted by the fact that KS tumors are highly vascularized even in early stages of development [4]. Multiple angiogenic cytokines including vascular endothelial growth factor (VEGF), basic fibroblast growth factor (b-FGF), angiopoietin-2, angiogenin and cycloxigenase-2 (COX-2) are induced during KSHV infection [5–13].

EGFL7, also known as a vascular endothelial (VE) statin, belongs to the epidermal growth factor-like domain family of growth factors. This angiogenic factor is secreted by the endothelial cells and highly upregulated in epithelial and endothelial tumors [14]. In addition to its role in promoting angiogenesis, EGFL7 has also been implicated in the growth and metastasis of many solid tumors [15–21]. The expression of EGFL7 is primarily restricted to endothelial cells with a very limited expression in most other human tissues; however, under pathological conditions such as cancer, many tumors cells are known to secrete EGFL7 in order to promote angiogenesis [19–21]. Interestingly, hypoxic conditions such as KS tumors are known to induce the expression of EGFL7, further indicating that EGFL7 plays an important role in angiogenesis [14, 22]. KS tumors, which show extensive neo-angiogenesis, are latently infected with KSHV and express latent proteins crucial for maintaining viral latency. Here, we explored the role of one of the predominant latent proteins, LANA in promoting cell growth and angiogenesis.

Latency-associated nuclear antigen [23] is one of the highly expressed latent proteins detected in all the infected cells [24–26]. LANA is a multifunctional protein that is essential for establishing and maintaining a successful latency [27]. LANA has been shown to promote angiogenesis and endothelial cell proliferation through the manipulation of multiple signaling pathways [28–30]. For example, LANA can promote angiogenesis by inducing the expression of VEGF A, an important angiogenic cytokine by stabilizing hypoxia-induced factor 1ɑ (HIF 1ɑ), a transcription factor that upregulates VEGF A expression [4, 11, 31, 32]. LANA also promotes angiogenesis by inhibiting the degradation of Hey1, a key component of the Notch signaling pathway with pronounced angiogenic activities [33]. LANA regulates the expression of many viral and cellular genes through its interactions with multiple host and viral proteins [1, 34, 35]. One such host protein that LANA interacts with is death domain-associated protein 6 (Daxx), a repressor of avian erythroblastosis virus E26 oncogene homolog 1 (Ets-1) [36]. Ets-1 is a transcription factor that positively regulates the expression of EGFL7 [14, 37]. Daxx is known to inhibit the transcriptional activity of several Ets-1 responsive promoters through direct protein-protein interactions [36, 38]. Here, we show that LANA expressing cells induce the expression of multiple growth and angiogenesis promoting genes including EGFL7. We identified the minimal promoter region of EGFL7, modulated by LANA, which contains the Ets-1 binding site. The upregulation of EGFL7 was attributed to LANA’s ability in sequestering Daxx and the subsequent removal of inhibition posed on Ets-1 bound to EGFL7 promoter. The role of EGFL7 in angiogenesis was confirmed by depleting the levels of EGFL7 using anti-EGFL7 siRNA in LANA expressing cells, which showed reduced *in vitro* tubule formation. Furthermore, scavenging the secreted EGFL7 from the supernatants of LANA expressing cells exhibited reduced tubulogenesis confirming EGFL7’s role in angiogenesis. Overall, KSHV infected cells have higher levels of EGFL7, which is upregulated by LANA to promote cell growth and angiogenesis.

## RESULTS

### EGFL7 was identified as one of the LANA regulated genes

**Figure 1:**
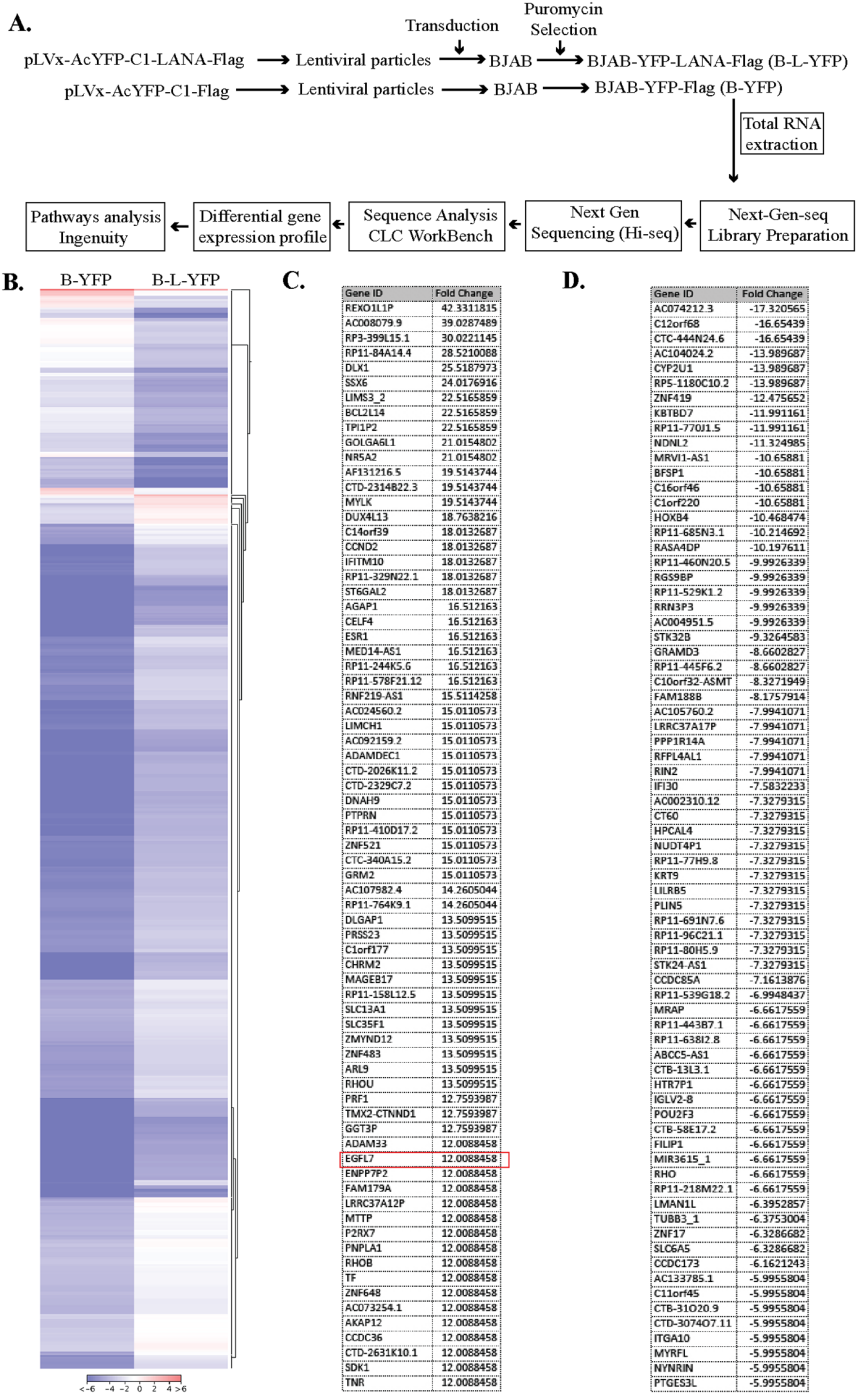
LANA expression modulates cellular gene expression **(A)** Experimental design for the RNAseq analysis of the LANA-expressing BJAB cells. The lentiviral particles expressing LANA, pLVx-AcYFP-C1-LANA-Flag and a control vector, pLVx-AcYFP-C1-Flag were used for transducing KSHV-negative BJAB cells. These cells were selected with puromycin to obtain a pure population of cells expressing target protein. The total RNA was extracted and used for RNAseq analysis on Illumina Hiseq. The sequence reads were analyzed for differential gene expression profile using CLC Workbench. LANA modulated genes were analyzed for pathways analysis using Ingenuity software (Qiagen, Inc.). **(B)** Heat-map to show differential gene expression. Genes with >5-fold changes (+) were used in this heat-map analysis by CLC Workbench. **(C)** List of upregulated genes (top 73) with EGFL7 encircled in red. **(D)** List of downregulated genes (top 73).

**Figure 2:**
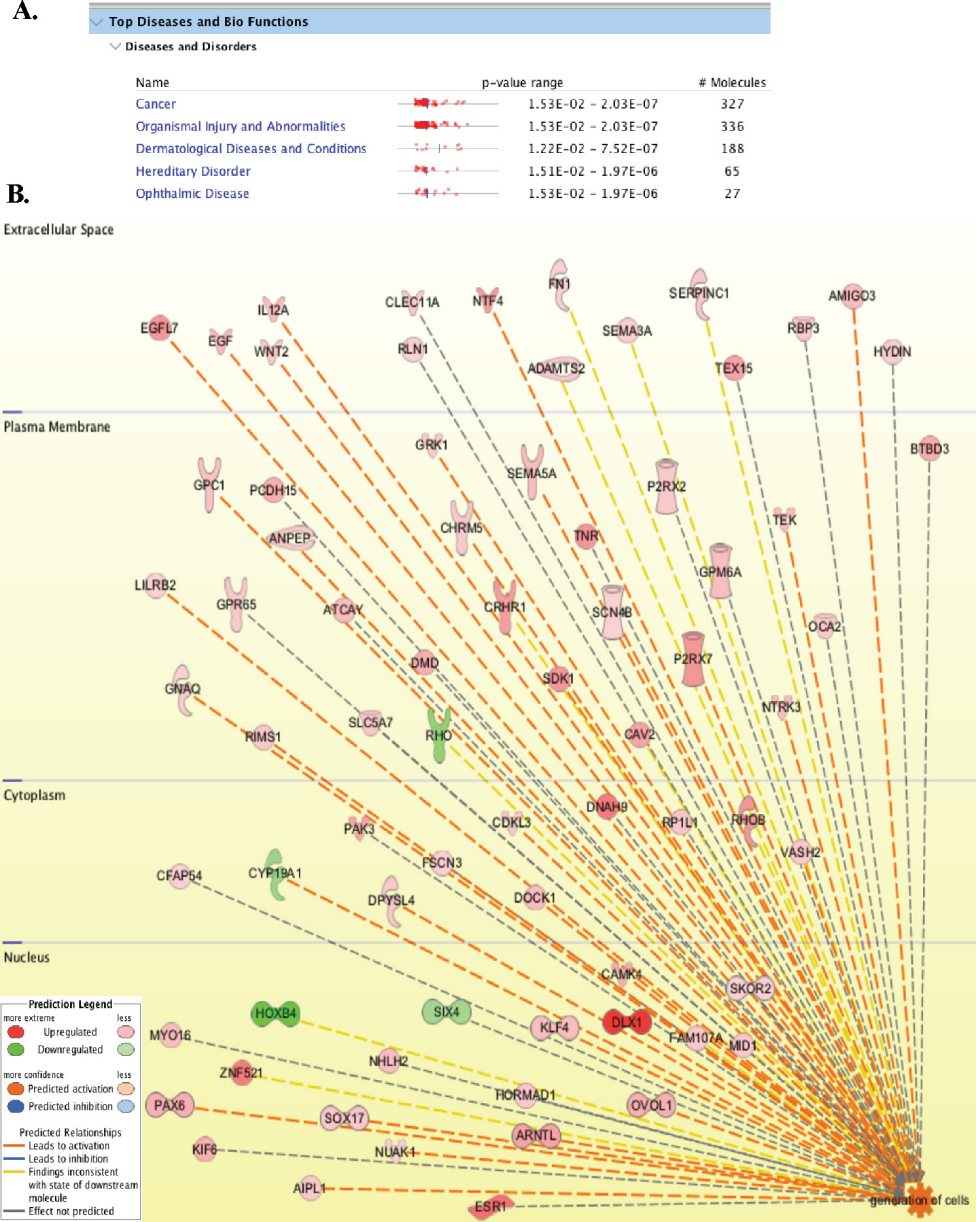
LANA modulated genes are involved in cancer, cell growth and angiogenesis **(A)** List of major disease pathways regulated by LANA in BJAB cells. **(B)** 630 molecules (cellular proteins) with >5-fold changes in LANA expressing cells were analyzed using Ingenuity pathways analysis tool for determining the global pathways affected by LANA. Among these pathways ‘generation of cells’ was highly activated by LANA. The upregulated genes are shown in red and the downregulated are in green. Genes activating the pathway are connected with orange color lines.

Considering the pleiotropic effects of LANA during KSHV latency, we wished to identify the host genes that were directly or indirectly regulated by LANA using a sensitive and unbiased approach of next-generation RNA sequencing. In order to identify the genes that were differentially regulated in response to LANA expression, we generated a LANA-expressing stable cell line (BJAB-L-YFP) and its matching control (BJAB-YFP) by transducing a KSHV-negative B cell line, BJAB with the lentivirus particles expressing the gene of interest. The expression of LANA was confirmed at mRNA (RT-PCR) and proteins levels (Western blotting) in these stably selected BJAB-L-YFP cells. These cells were used for the identification of LANA regulated genes through the steps shown in a schematic (Figure 1A). Briefly, total RNA extracted from these cells were used for preparing the cDNA libraries for RNA sequencing using Illumina HiSeq. The sequenced data was mapped to the reference human genome (hg19) for relative expression of cellular genes using CLC Workbench. We identified a large number, both positively and negatively regulated, genes in BJAB cells expressing LANA. A list of genes modulated (both up and down regulated) by more than 5 folds is presented as Supplementary Table 1. A representation of differential gene expression in LANA expressing cells is shown as a heatmap (Figure 1B). The lists of significantly upregulated and down regulated genes are presented in Figure 1C and 1D, respectively. Analysis of these genes using a pathway analysis tool, Ingenuity, showed that the majority of LANA modulated genes are involved in cancer related pathways (Figure 2A). To determine the effect of these genes on specific pathways, we analyzed these molecules for their combined effect, which identified ‘generation of cells’ as an important upregulated pathway in LANA expressing cells (Figure 2B). Among these significantly upregulated genes, EGFL7 was selected for further study considering its role in cell growth and angiogenesis, which are considered important in KSHV induced malignancies.

### EGFL7 was upregulated in KSHV infected cells and tissues

**Figure 3:**
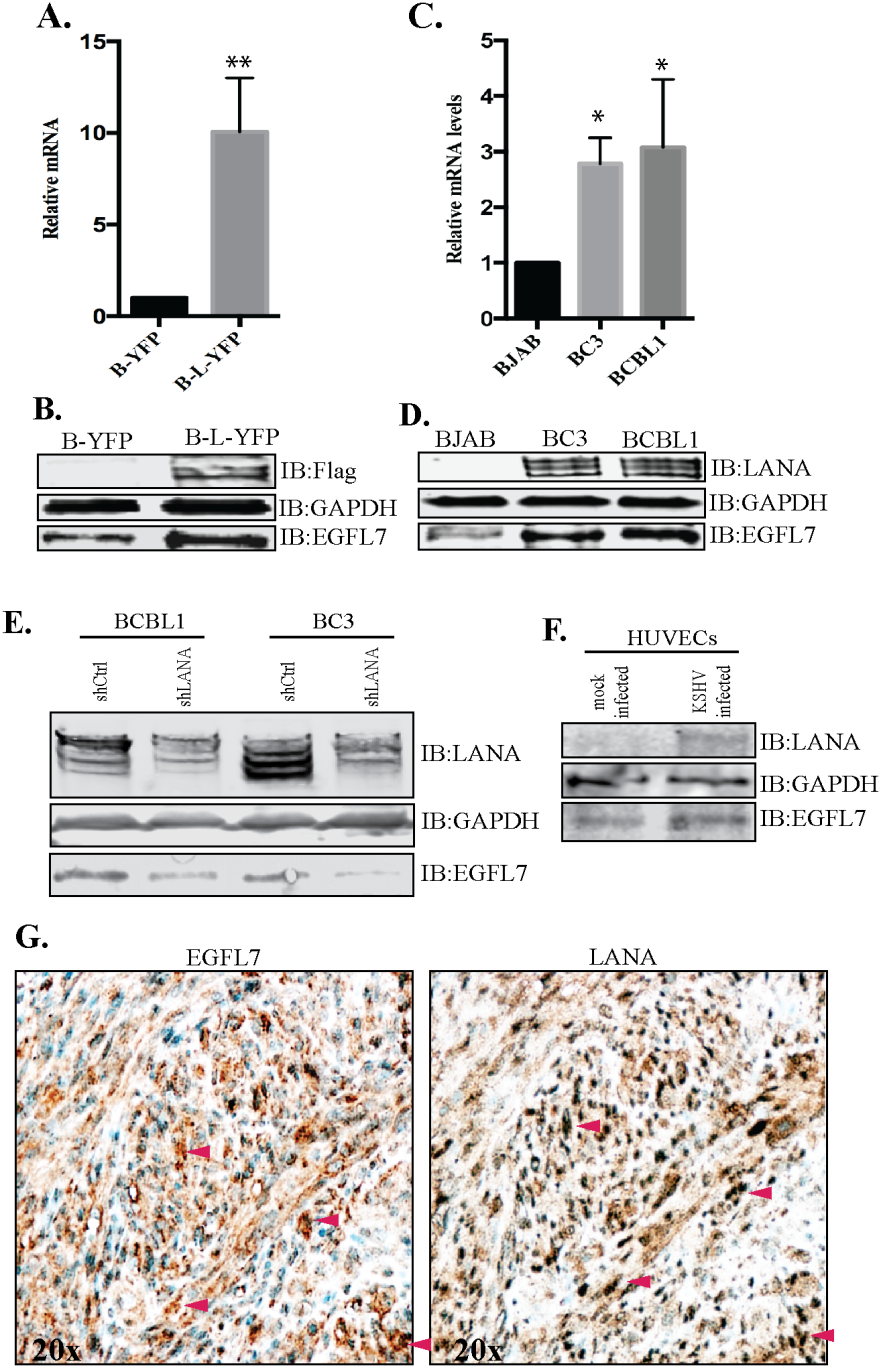
EGFL7 was upregulated in LANA expressing BJAB and KSHV infected PELs and KS-tissues **(A)** BJAB cells stably expressing LANA (B-L-YFP) were analyzed for relative mRNA levels of EGFL7, which showed a significantly higher level as compared to the control BJAB (B-YFP) cells. The mRNA levels were quantified using RT-PCR and the expression levels were normalized against the GAPDH gene. The error bars represent standard deviations from the mean of at least three experimental replicates. ^**^ Indicates P < 0.005. **(B)** Immune detection of EGFL7 protein in LANA expressing (B-L-YFP) and control (B-YFP) BJAB cells, which showed relatively higher levels of EGFL7 in LANA expressing cells. Expression of LANA was detected using anti-Flag and GAPDH was used as a loading control. **(C)** KSHV infected cells, BC3 and BCBL1 showed a higher level of EGFL7 mRNA as compared to KSHV negative, BJAB cells. The mRNA levels were quantified using RT-PCR and the expression levels were normalized against the GAPDH gene. The error bars represent standard deviations from the mean of at least three experimental replicates. ^*^ Indicates P < 0.05. **(D)** KSHV infected cells, BC3 and BCBL1 have higher levels of EGFL7 proteins detected in a western blot. LANA was detected using rabbit anti-LANA antibody and GAPDH was used as loading control. **(E)** LANA depleted KSHV infected, BCBL1 and BC3 cells were analyzed for EGFL7 levels. The shLANA transduced BCBL1 and BC3 cells showed a significantly reduced level of EGFL7 as compared to the control, shCtrl cells. LANA immunoblot was used for the detection of LANA levels in these shRNA transduced cells, which showed a significantly reduced levels in LANA specific shRNA (shLANA) cells as compared to the control, shCtrl cells. GAPDH was used as loading control. **(F)** HUVECs were infected with purified KSHV virions to detect the levels of EGFL7 during *de novo* infection. Cell lysates from the KSHV infected HUVECs (72hpi) showed an increase in the levels of EGFL7 as compared to the mock infected cells. LANA was detected in immunoblot as expected. GAPDH was used as a loading control. **(G)** Higher levels of EGFL7 correlated with LANA expression in KS tissue detected in immunohistochemistry (IHC). Red arrows indicate corresponding cells in both panels.

Following the identification of EGFL7 as a LANA upregulated gene in RNAseq analysis, we wanted to confirm the levels of EGFL7 using independent approaches including RT-PCR and Western blot detection. As expected, the relative mRNA levels of EGFL7 were considerably higher in the LANA-expressing BJAB (B-L-YFP) cells as compared with the control BJAB (B-YFP) cells (Figure 3A). Immune detection of proteins levels in BJAB expressing LANA (B-L-YFP) showed an upregulation in EGFL7 compared to the control (B-YFP) cells (Figure 3B). Expression of YFP-fused-LANA tagged with flag epitope was detected using anti-flag antibody. GAPDH served as a loading control. Consistent with the observed increase in EGFL7 transcript level, EGFL7 protein was also increased in LANA-expressing cells compared with the control cells (Figure 3B).

RNAseq and RT PCR data confirmed that the expression of LANA was sufficient to induce the expression of EGFL7, next we wanted to determine whether KSHV infected cells have an elevated level of EGFL7. To this end, we analyzed the EGFL7 mRNA levels in KSHV-infected primary effusion lymphoma cell lines, BCBL1 and BC3 and compared with a KSHV negative cells line, BJAB. The relative abundance of the EGFL7 transcript was significantly higher in both the KSHV-positive cell lines (BC3 and BCBL1) compared to the KSHV-negative BJAB cells (Figure 3C). The upregulation of EGFL7 protein was also detected in KSHV infected primary effusion lymphoma cells (BC3 and BCBL1) compared to the control, BJAB cells (Figure 3D). Significant upregulation of EGFL7 in primary effusion lymphoma cell lines suggest that EGFL7 likely plays an important role in KSHV induced malignancies.

To ensure whether LANA was contributing in increasing the levels of EGFL7, we depleted LANA from the KSHV infected BCBL1 and BC3 using shRNA as described previously [39]. Our data showed a partial depletion of LANA in shLANA BCBL1 and BC3 cells when compared to the control shRNA (shCtrl) cells (Figure 3E). Importantly, the levels of EGFL7 in these cell lysates were significantly reduced in LANA depleted cells as compared to the control (shCtrl) cells (Figure 3E), confirming the role of LANA in regulating EGFL7 expression. We also tested the levels of EGFL7 during *de novo* infection of KSHV into the target cells, HUVEC (human unbilical vein endothelial cells). Purified KSHV virions were used for infecting the HUVECs as described previously [40] and the cells from 72 hours post-infection (hpi) and mock infection were collected for the detection of EGFL7 levels. Our data showed a detectable level of LANA and significantly increased levels of EGFL7 in KSHV infected HUVECs as compared to the mock infected HUVECs (Figure 3F). These data confirmed that the KSHV infected cells have a higher level of EGFL7 due to LANA expression.

Analysis of multiple KS biopsy samples including skin and lymph nodes showed strong correlation of high levels of EGFL7 expression with LANA detection. A representative image of skin biopsy sample is shown in Figure 3G. These data confirmed that KSHV infected cells have higher levels of EGFL7, which may contribute to an enhanced cellular progression and angiogenesis.

### LANA upregulates EGFL7 promoter activity

**Figure 4:**
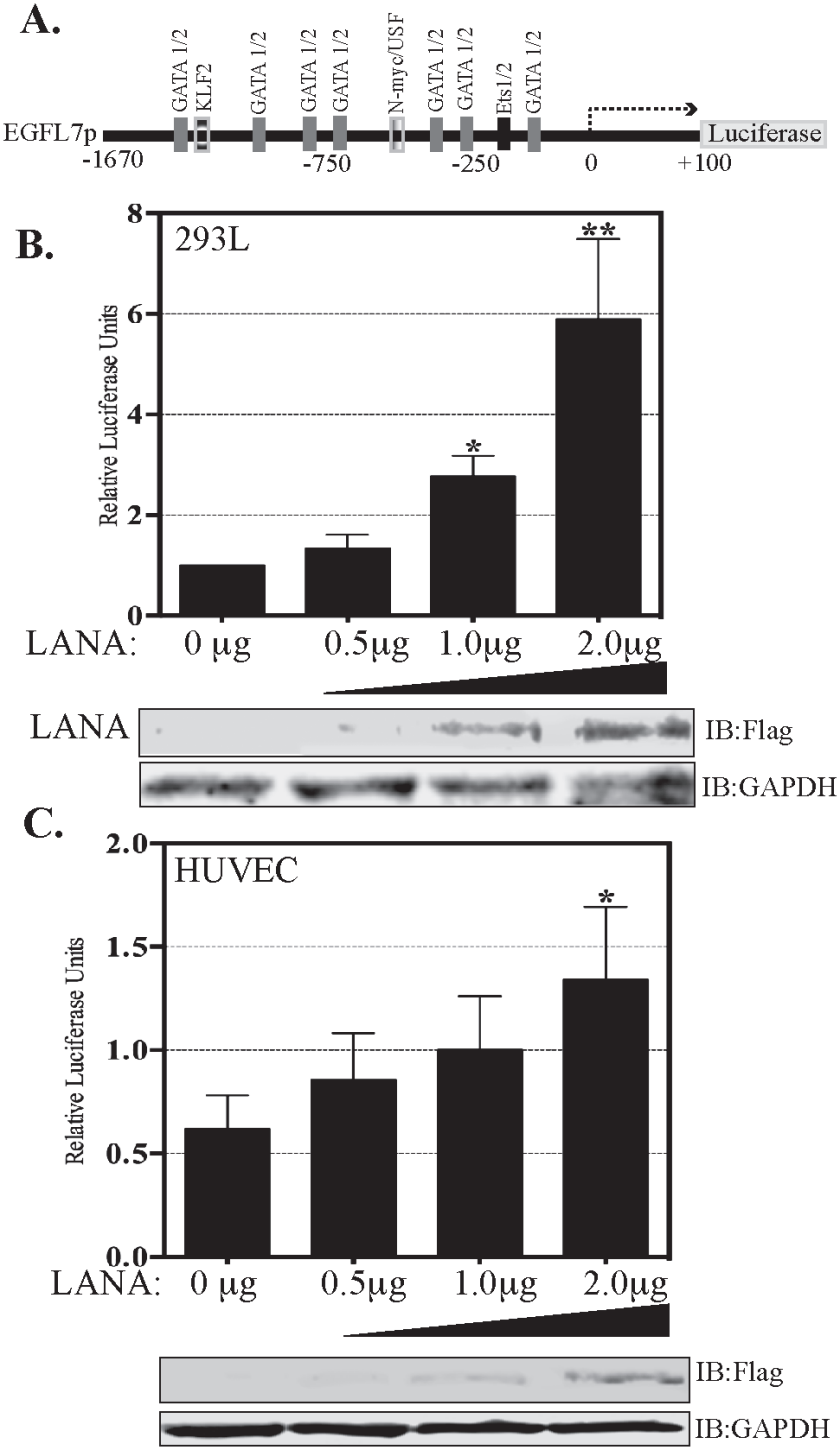
LANA activates EGFL7 promoter **(A)** Schematic of full-length EGFL7 promoter-luciferase construct showing a map of the transcription factor binding sites. The dual luciferase reporter assay results show a dose-dependent increase in EGFL7 luciferase activity in response to an increasing expression of LANA. **(B)** HEK293L cells and **(C)** HUVEC cells. The cells were transfected with 0.5 μg, 1.0 μg or 2.0 μg of Flag-tagged LANA-expressing plasmid pA3F-LANA. RLU were calculated relative to the promoter activity of 0 μg LANA. The error bars represent the standard deviations from the mean of at least three replicates. ^*^ Indicates P < 0.05 and ^**^ Indicates P < 0.001. Lysate were used for the detection of LANA using anti-Flag antibody and GAPDH was used as control for equal lysates used in the assay.

LANA has been shown to modulate the activities of many cellular and viral promoters. Hence, we examined whether LANA enhances the expression of EGFL7 by activating the EGFL7 promoter. To this end, we performed promoter reporter assays using a full-length EGFL7 promoter luciferase construct [41]. The EGFL7 promoter contains the binding sites of multiple transcription factors depicted as schematic (Figure 4A). The reporter assays performed in two different cell lines, HEK293L cells (Figure 4B) and endothelial, HUVEC cells (Figure 4C) showed LANA mediated upregulation of full-length EGFL7 promoter (-1670 to +100), presented as relative luciferase units, in a dose-dependent manner (Figure 4B and 4C). These results confirmed that LANA induces the expression of EGFL7 by activating its promoter. Expression of LANA-Flag in these reporter assays was detected using anti-Flag antibody in the lysates used for the luciferase assay. GAPDH served as loading control.

### LANA upregulates EGFL7 expression by sequestering Daxx

**Figure 5:**
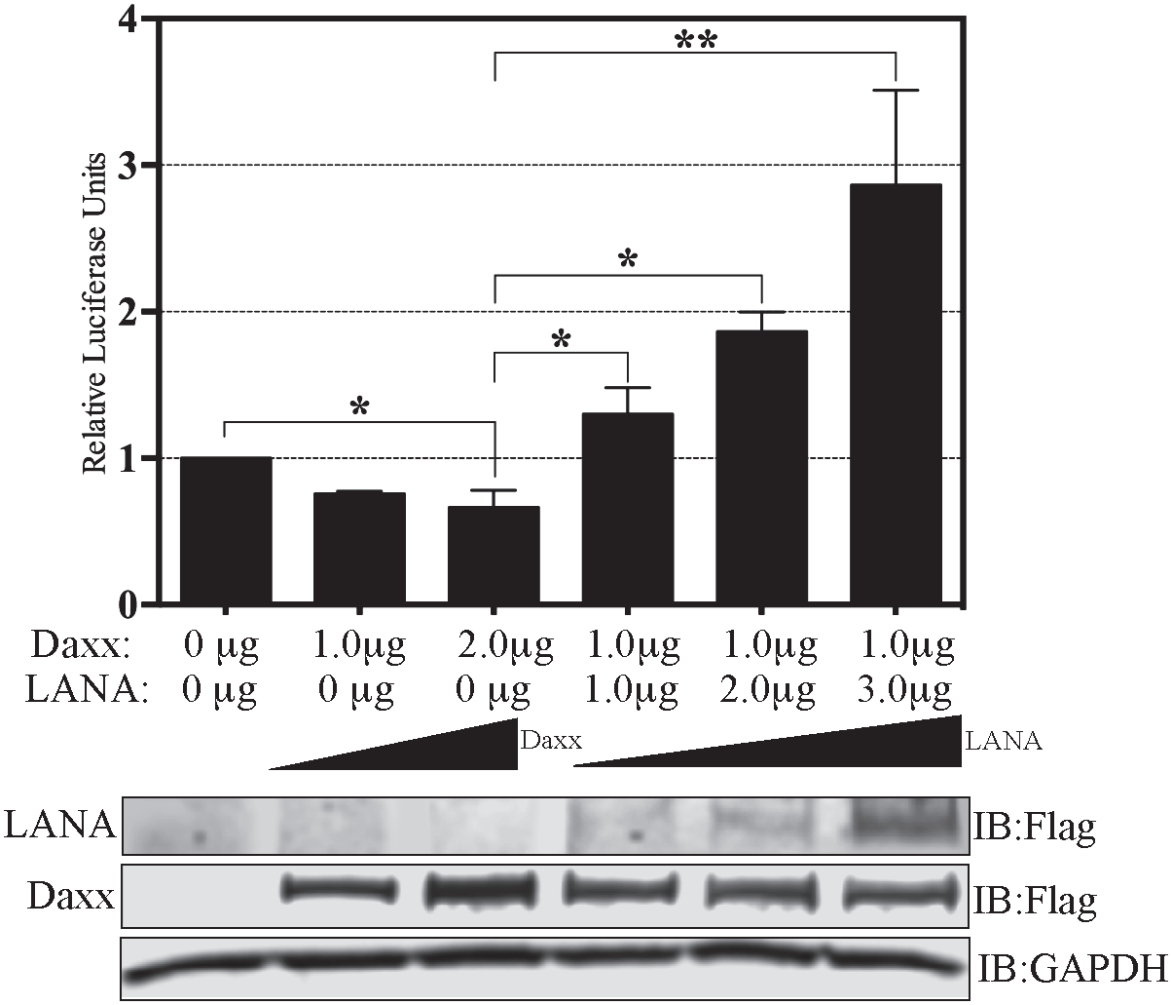
LANA upregulates EGFL7 promoter by sequestering Daxx Dual luciferase reporter assay in HUVEC cells in presence of Daxx and LANA. Relative Luciferase Units were calculated relative to the promoter only activity. HUVEC cells transfected with 1.0 μg or 2.0 μg of Daxx showed repression in the EGFL7’s promoter activity as compared to the promoter only lane. Expression of 1.0 μg of Daxx repressed EGFL7 activity but a co-expression of LANA rescued the promoter activity in a dose dependent manner. The error bars represent standard deviations from the mean of at least three replicates. ^*^ Indicates P < 0.05 and ^**^ Indicates P < 0.001. Expression of Daxx and LANA was detected using anti-Flag antibody and GAPDH was used a control for equal loading of the lysates.

LANA can potentially activate promoters in multiple potential ways such as: increasing the expressions of one or more transcription factors necessary for EGFL7 expression, recruitment of activators, or the removal of repressors of the EGFL7 promoter. Since previous studies have demonstrated that LANA sequesters Daxx, which acts as a repressor of the promoter activity by inhibiting Ets-1, and the EGFL7 promoter has the Ets-1 binding site, we speculated that this mechanism might be contributing to the activation of EGFL7 promoter by LANA. In order to test this hypothesis, we performed a series of reporter assays in HUVEC cells transfected with Daxx and/or LANA (Figure 5). As expected, the overexpression of Daxx reduced the activity of EGFL7 promoter (Figure 5, compare lanes containing 1 and 2 μg of Daxx to lane without Daxx), confirming that Daxx represses the EGFL7 promoter. Increasing amounts of Daxx were unable to repress the promoter activity in a dose dependent manner although this may be due to the saturating levels of repression by endogenous Daxx. However, consistent with our hypothesis, expression of LANA alleviated the repressive effects of Daxx mediated repression in a dose dependent manner (Figure 5 lanes with 1.0, 2.0 and 3.0 μg of LANA). The increase in the promoter activity with 3.0 μg of LANA was higher than the cells without Daxx suggesting that the excess expression of LANA not only sequestered overexpressed Daxx but also sequestered the endogenous Daxx. This confirmed that sequestration of Daxx is the primary mechanism responsible for the upregulation of EGFL7 promoter activity by LANA. Cellular lysates of these samples were used for the detection of overexpressed Daxx and LANA with anti-flag antibody (Figure 5). GAPDH was used as a loading control among these samples.

**Figure 6:**
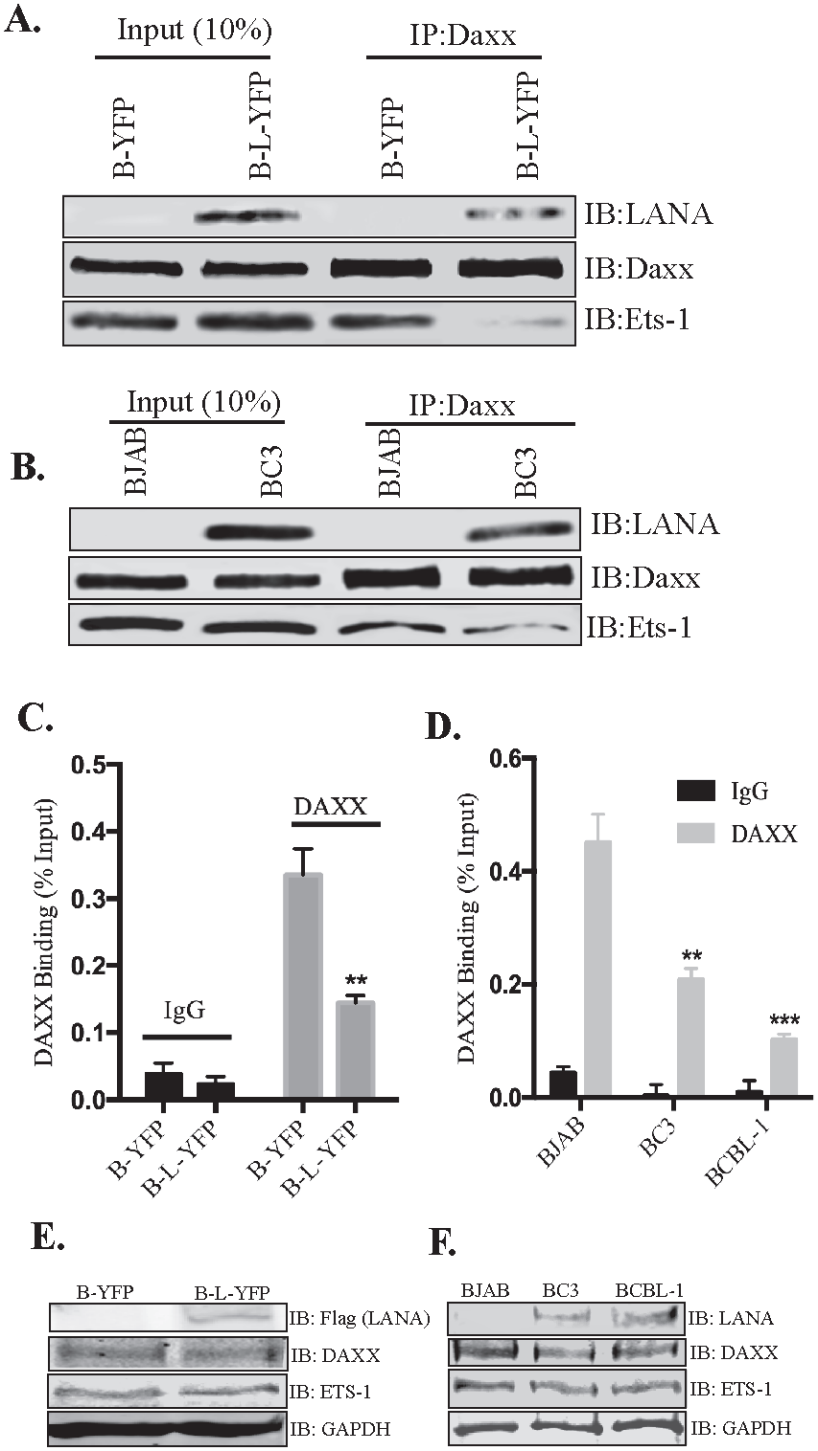
LANA reduced the binding of Daxx to Ets-1 and EGFL7 promoter **(A)** Immunoprecipitation of Daxx from LANA expressing (B-L-YFP) and control (B-YFP) BJAB cells. Detection of Ets-1 shows significantly lower amounts of co-precipitating Ets-1 from BJAB with LANA (B-L-YFP). Input showed comparable levels of Ets-1 in both, LANA and control BJAB cells. Co-precipitating LANA was also detected with Daxx in LANA expressing BJAB confirming their interactions. **(B)** KSHV infected BC3 and KSHV negative BJABs were subjected for Daxx immunoprecipitation for the detection of co-precipitating Ets-1, which showed lower levels in BC3 as compared to the BJAB cells. LANA was also detected co-precipitating with Daxx confirming their interaction. **(C)** Binding of Daxx to the chromatin of EGFL7 promoter was reduced in LANA expressing cells. Chromatin immunoprecipitation (ChIP) performed with anti-Daxx antibody and the detection of EGFL7 promoter in a real-time PCR assay. Relative binding of Daxx to the EGFL7 promoter was determined in LANA expressing (B-L-YFP) and control BJAB (B-YFP) cells and are presented as a percentage of the respective input samples. Binding of Daxx to the promoter in LANA expressing cells (B-L-YFP) was significantly reduced as compared to the control cells. Enrichment of EGFL7 compared to the matched IgG control confirmed the specificity of this ChIP assay. **(D)** Relative binding of Daxx to the EGFL7 promoter was determined in KSHV infected cells, BC3 and BCBL1 as compared to the KSHV negative, BJAB cells. The data presented, as the percentage of respective inputs, showed a significantly reduced binding of Daxx in BC3 and BCBL1 cells as compared to the BJAB. Enrichment of EGFL7 compared to the matched IgG control confirmed the specificity of this assay. ^*^ Indicates P < 0.05 and ^**^ Indicates P < 0.001. **(E)** Immune detection of Daxx and Ets-1 in the lysates of LANA expressing (B-L-YFP) and control (B-YFP) BJABs. Expression of LANA was detected by flag epitope tag of LANA-YFP. GAPDH was detected for loading control. **(F)** Immune detection of Daxx and Ets-1 in the lysates of BJAB, BC3 and BCBL1 using specific antibodies. LANA was detected using anti-LANA antibody in BC3 and BCBL1 cells. GAPDH was used as a loading control.

In the absence of LANA, Daxx associates with Ets-1 in order to repress its activity but LANA’s binding with Daxx disrupts its association with Ets-1 by sequestering Daxx away from the promoter [36]. Using co-immunoprecipitation assays, we confirmed that the association of Ets-1 with Daxx was indeed reduced in the presence of LANA (Figure 6A). Immunoprecipitation of Daxx from BJAB cells expressing LANA showed considerably lower amounts of co-precipitating Ets-1 as compared to the control BJAB cells (B-YFP). Co-precipitated LANA was also detected in LANA expressing BJAB cells (Figure 6A, B-L-YFP lane). There was comparable amount of Daxx precipitating with anti-Daxx antibody in both, LANA expressing and control, cell lines. LANA mediated disruption of Daxx binding to Ets-1 was also confirmed in KSHV infected cells (Figure 6B). Immunoprecipitation of Daxx from BC3 cells showed significantly reduced amounts of co-precipitating Ets-1 as compared to the KSHV negative, BJAB cells (Figure 6B). Binding of LANA to Daxx was also confirmed by the detection of LANA in Daxx immunoprecipitated samples from BC3 cells. Comparable amounts of Daxx were detected in the input and IP samples of BJAB and BC3 cells.

Furthermore, LANA-mediated removal of Daxx from the EGFL7 promoter was confirmed using ChIP assay (Figure 6C and 6D). Immunoprecipitation of chromatin with anti-Daxx antibody from LANA-expressing BJAB (B-L-YFP) and the control cells (B-YFP) showed specific binding of Daxx to the EGFL7 promoter, which was significantly reduced in cells expressing LANA (Figure 6C). Matched IgG control was used in the ChIP assay, which showed minimal binding thus confirming the specificity of Daxx binding to the EGFL7 promoter (Figure 6C). The levels of Daxx binding to the EGFL7 promoter were also compared in KSHV infected cells, BC3 and BCBL1 cells with a KSHV negative, BJAB cells (Figure 6D). The data showed reduced binding of Daxx to the EGFL7 promoter in KSHV infected cells (Figure 6D) suggesting that LANA efficiently removes Daxx from the EGFL7 promoter. IgG control antibody showed minimal binding confirming a specific association of Daxx to the EGFL7 promoter (Figure 6D). Collectively, these data suggested towards the sequestration of Daxx by LANA as a primary mechanism behind LANA-mediated transactivation of the EGFL7 promoter. In order to ensure that the reduction in Daxx binding to the EGFL7 promoter was due to a lower level of Daxx in LANA expressing cells, we analyzed the lysates of control (B-YFP) and LANA expressing BJABs (B-L-YFP) for Daxx and Ets-1 (Figure 6E). Comparable levels of both Daxx and Ets-1 supported LANA mediated sequestration of Daxx from the EGFL7 promoter. Similarly, the levels of Daxx and Ets-1 were also comparable between KSHV negative, BJAB and positive, BC3 and BCBL1 cells (Figure 6F).

### The Ets-1 binding region of the EGFL7 promoter is responsible for LANA-mediated induction of EGFL7

**Figure 7:**
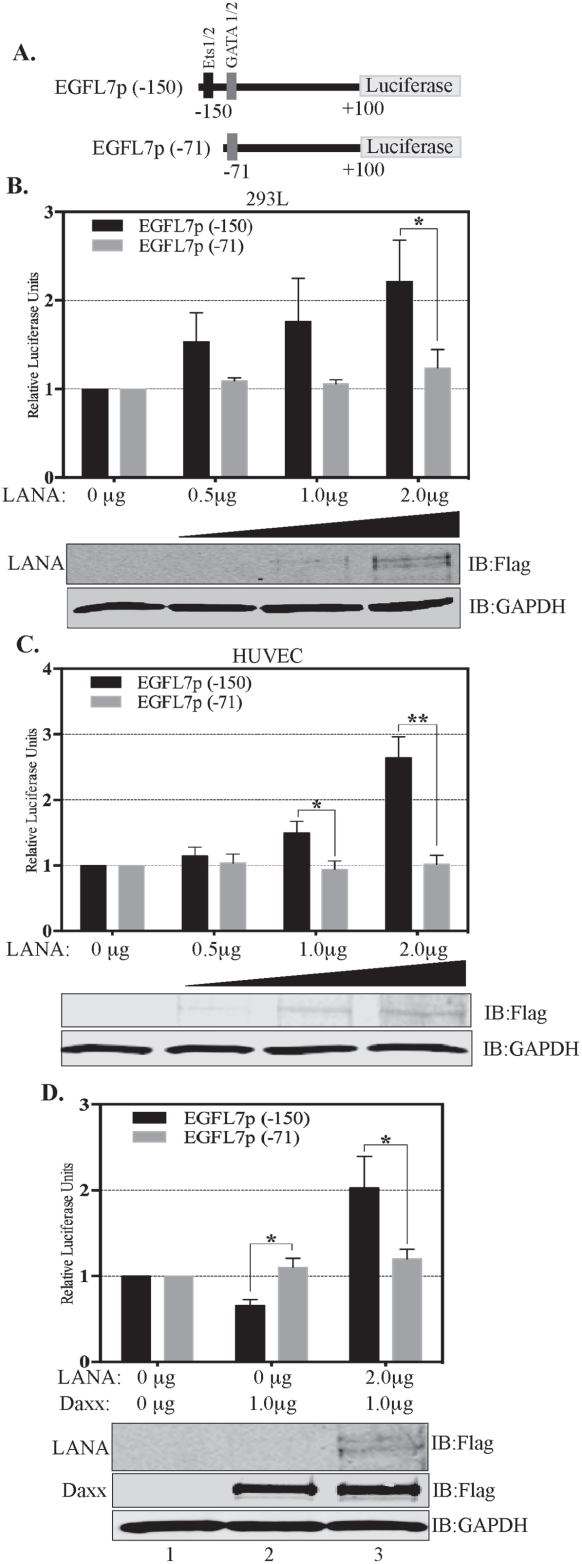
Ets-1 binding site is important for the transactivation of EGFL7 promoter by LANA **(A)** Schematic showing deletion constructs used in the dual luciferase assays in response to an increasing concentration of LANA. EGFL7p (-150) truncation contains the Ets-1 binding site but not the EGFL7p (-71) **(B)** HEK293L cells and **(C)** HUVEC cells. The concentrations of LANA expressing plasmids (Flag epitope tagged LANA) transfected in these cells are indicated below each bar. Increasing amounts of LANA enhanced the EGFL7 promoter activity in truncation with Ets-1 site (EGFL7p (-150) (black bars) but not in the truncation lacking Ets-1 site (EGFL7p (-71) (grey bars). LANA in corresponding lanes showed increasing expression detected by anti-Flag antibody. GAPDH was used as loading control. **(D)** Truncation of EGFL7p lacking Ets-1 binding site, EGFL7p (-71) did respond to Daxx or LANA expression confirming that LANA modulates EGFL7 promoter through Ets-1. The black bars represent relative luciferase activities of the (-150 to +100) construct and the grey bars represent the relative luciferase activities of the (-71 to +100) construct. RLU were calculated relative to the promoter only cells. The error bars represent the standard deviations from the mean of at least three replicates. ^*^ Indicates P < 0.05 and ^**^ Indicates P < 0.001. Expressions of LANA and Daxx were detected using anti-Flag antibody. GAPDH was used as a control for equal amounts of lysates used in the assay.

To further confirm that Daxx sequestration is the primary mechanism responsible for the transactivation of the EGFL7 promoter by LANA, we studied the regulation of EGFL7 promoter in response to LANA using deletion analysis of the EGFL7 promoter. The EGFL7 promoter contains potential binding sites for several transcription factors including the binding sites for GATA1, GATA2, Ets-1 and Ets-2 (Figure 4A) [41]. Of these, the EGFL7 promoter region -150 to + 100 contains binding sites for Ets-1 and 2, as well as for GATA 1 and 2; and the region -71 to + 100 contains binding sites for GATA 1 and 2 only. We generated these two deletion constructs of the EGFL7 promoter (-150 to +100 and -71 to +100) for deciphering the role of Ets-1 binding site regulating EGFL7 promoter (Figure 7A).

Reporter vector with these two truncations was transfected into 293L (Figure 7B) and HUVEC (Figure 7C) cells with and without LANA. The data of these experiments revealed that the region -150 to +100 of EGFL7 promoter was transactivated with LANA in a dose-dependent manner (black bars, Figure 7B and 7C) but not the-71 to +100 region (white bars, Figure 6B and 6C) in both, HEK293L and HUVEC cells (Figure 7). The promoter region (-150 to +100), which responded to the LANA expression, contains Ets-1 binding site confirming its role in promoter activation. The specificity of Daxx in repressing the EGFL7 promoter through Ets-1 binding was demonstrated by determining the responsiveness of -150 to +100 and -71 to +100 promoter regions due to overexpression of Daxx and LANA together (Figure 7D). Expression of Daxx inhibited the activity of the -150 to +100 promoter reporter vector containing the Ets-1 binding site (Figure 7D, lane 2, black bar) but not the -71 to +100 promoter reporter vector, which lacks the Ets-1 binding site (Figure 7D, lane 2, grey bar). Furthermore, LANA expression significantly increased the promoter activity of -150 to +100 construct but not the -71 to 100 construct, indicating that LANA cannot activate the EGFL7 promoter without the Ets-1 binding site (Figure 7D, lane 3). Taken together, these results clearly demonstrate that a sequestration of Daxx is the primary mechanism by which LANA upregulates EGFL7 transcription.

### EGFL7 contributes to LANA-induced angiogenic phenotype *in vitro*

**Figure 8:**
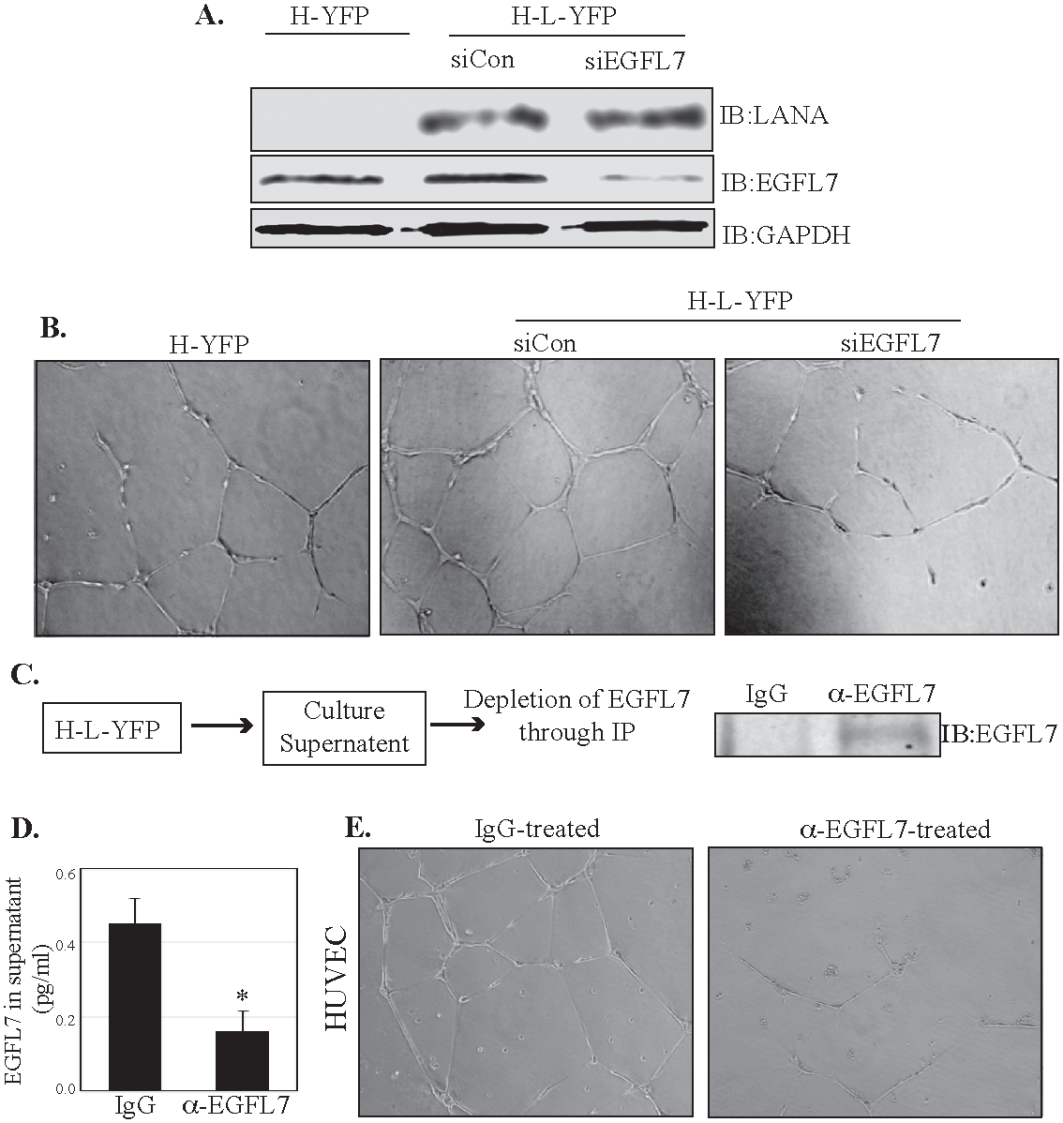
EGFL7 contributes to the LANA-induced *in vitro* endothelial tubule formation **(A)** HUVEC cells stably expressing LANA-YFP (H-L-YFP) or YFP (H-YFP) were used for the detection of EGFL7 levels, which was significantly higher in LANA expressing cells (H-L-YFP si Con) as compared to the YFP expressing cells (H-YFP). Cells with siCon were used as a LANA expressing cells as the control siRNA did not have any effect on EGFL7 expression. EGFL7 siRNA significantly depleted the levels of EGFL7. LANA was detected in HUVECs with LANA-YFP and GAPDH was used as a loading control. **(B)** These cells were plated on Geltrex Matrix-coated plates and imaged after 24 h of plating. Representative images of tubule formation in control HUVECs (H-YFP), LANA expressing siCon HUVECs (H-L-YFP siCon) and LANA expressing siEGFL7 HUVECs (H-L-YFP siEGFL7). EGFL7 depleted HUVECs (H-L-YFP siEGFL7) showed a reduced tubulogenesis as compared to the siCon HUVECs. **(C)** Culture supernatants (5ml) from LANA expressing HUVECs were collected and subjected to the depletion of EGFL7 using anti-EGFL7 antibody or control, IgG antibody. Immunoprecipiated EGFL7 from the supernatants was detected by Western blot. **(D)** The residual levels of EGFL7 were detected in the supernatant treated with anti-EGFL7 or control, IgG antibody using an ELISA assay. **(E)** Supernatants treated with anti-EGFL7 or control antibody (IgG) were used on HUVEC cells for tubulogenesis assay. Representative image show that EGFL7 depleted supernatants had significantly reduced tubule formation as compared to the control antibody (IgG) treated supernatants. This confirmed the role secreted EGFL7 in tubulogenesis.

LANA has been demonstrated to promote angiogenesis through a variety of mechanisms [29, 33, 36, 42, 43]. EGFL7 also has been shown to promote angiogenesis in a variety of cancers [14]. We therefore investigated whether EGFL7 contributes to LANA-induced angiogenesis. To this end, we analyzed the angiogenesis of HUVEC cells expressing LANA and compared it with the control cells using an *in vitro* endothelial tube formation assay (Figure 8). The role of EGFL7 was determined by depleting the EGFL7 levels using siRNA in the HUVEC cells stably expressing LANA. EGFL7 depleted LANA expressing HUVEC cells showed decreased tubule formation (determined by the number of branches) as compared to the cells with control siRNA (Figure 8B). Importantly, HUVEC with LANA-YFP showed higher levels of tubulogenesis as compared to the vector, YFP cells (Figure 8B). Protein expressions showed enhanced EGFL7 expression in LANA-YFP cells and reduced levels in siEGFL7-depleted cells, as expected (Figure 8A), confirming that enhanced tubulogenesis was due to LANA-mediated upregulation of EGFL7 expression. We were further interested in determining whether the secreted EGFL7 plays an important role in enhancing angiogenesis on surrounding cells in a paracrine mechanism. To this end, we collected the supernatants from HUVEC cells expressing LANA (H-L-YFP) and scavenged the EGFL7 from the supernatant by immunoprecipitating with EGFL7 specific or isogenic control antibody (Figure 8C). Immunoprecipitation of secreted EGFL7 was confirmed in a Western blot and the levels of remaining EGFL7 were detected using ELISA assay (Figure 8C), which showed a significantly reduced level in α-EGFL7 supernatants as compared to the control IgG sample (Figure 8D). The antibody stripped supernatants were used on HUVEC cells for tubulogenesis assay. Culture supernatant with depleted EGFL7 showed limited tubule formation as compared to the control antibody depleted supernatant confirming that EGFL7 acts in a paracrine mechanism to enhance angiogenesis (Figure 8E). This confirmed that LANA upregulated EGFL7 contributes to tubulogenesis through autocrine and paracrine mechanisms.

## DISCUSSION

KSHV LANA is the most abundantly expressed protein during latency, which regulates multiple cellular pathways by modulating promoter activities and protein-protein interactions [1, 27]. Expression of LANA in KSHV and EBV negative B cells, BJAB altered the expression of multiple cellular proteins. A large number of these molecules were related to cancer inducing or cancer maintenance genes, confirming LANA is an oncogenic protein. Global effects of these modulations led us to identify multiple pathways important in the progression of cellular growth, generation of cells, and angiogenesis. EGFL7 was identified as one of the upregulated genes in our screen designed to identify differentially regulated LANA responsive cellular genes using next-generation RNA sequencing analysis. EGFL7 activates pathways involved in generation of cells and angiogenesis. Angiogenesis is a critical process for the survival of tumor cells since it fulfills the increased demands of oxygen and nutrients required by the rapidly dividing tumor cells. As a result, many solid tumors induce the expression of pro-angiogenic cytokines. EGFL7 is one such angiogenic cytokine overexpressed in multiple malignancies such as malignant glioma, colorectal cancer, renal cell carcinoma and hepatocellular carcinoma [15–21]. In these tumors, EGFL7 has been found to be positively associated with angiogenesis, growth and invasiveness. Extensive angiogenesis is an important feature of KS tumors. In light of the role EGFL7 has in other tumors that require extensive angiogenesis, it most likely plays an important role in the progression of KS tumors as well.

**Figure 9:**
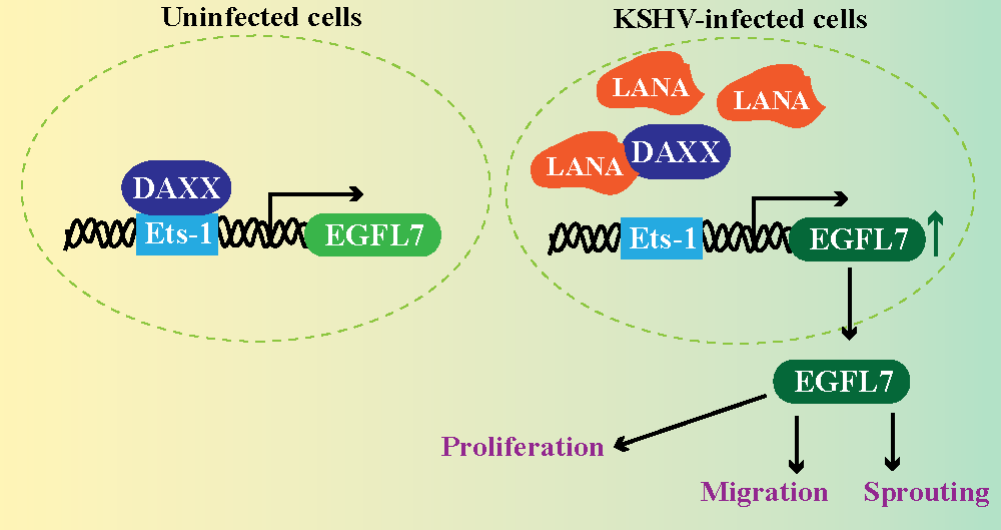
LANA upregulates EGFL7 expression by sequestering Daxx bound with Ets-1 at the promoter Normal cells exhibit binding of Daxx to the Ets-1 promoter to repress the expression of EGFL7. However, KSHV infected cells expresses LANA, which binds to Daxx and sequesters it from the Ets-1 bound to the EGFL7 promoter. This alleviates the repressive effects caused by Daxx leading to an enhanced expression of EGFL7. EGFL7 can modulate various cellular pathways to promote cell proliferation, cell migration and sprouting of blood vessels important for angiogenesis.

Hypoxia has also been shown to induce expression of EGFL7 [44]. The KS tumor environment is also highly hypoxic; KS tumors frequently arise in locations with low oxygen levels such as the lower extremities [22, 45, 46]. Based on these findings, we further investigated the role of EGFL7 during KSHV infection and the underlying mechanism in this study. Our results revealed that EGFL7 transcript and protein expression were significantly elevated in latently KSHV-infected primary effusion lymphoma cells, BC3 and BCBL1, compared to the KSHV-negative BJAB cells. The fact that LANA is responsible for this upregulation was highlighted from the next generation sequencing data, which showed elevated levels of EGFL7 transcripts in B cells expressing LANA as the sole KSHV protein. An intriguing aspect of these findings is the elevated expression of EGFL7 in B cells. Normally, B cells do not participate in angiogenesis; therefore, the significance of EGFL7 overexpression in B cells during KSHV infection points toward the possible involvement of the paracrine mechanism inducing angiogenesis in the spindle cells of KS lesions. EGFL7 expression is highly restricted to the endothelial cells but can express under certain physiological conditions that require angiogenesis such as embryogenesis and wound healing. This restricted pattern of EGFL7 expression could be at least part due to the inhibitory effects of Daxx protein on the Ets-1-dependent transactivation of the EGFL7 promoter [38]. The interaction of Daxx with Ets-1 has been previously reported to repress transcriptional activation of at least three genes involved in the promotion of angiogenic phenotypes (MMP1, Bcl2 and Flt-1/VEGF receptor 1) [36, 38]. Interestingly, LANA has been shown to sequester Daxx and consequently relieve Daxx-mediated inhibition on Ets-1-dependent VEGF receptor 1 promoter [36]. Because the EGFL7 promoter also contains binding sites for Ets-1, we investigated whether LANA could activate the EGFL7 promoter by sequestering Daxx in a similar fashion. Our results showed that LANA could indeed activate EGFL7 promoter even in the presence of Daxx, by displacing Daxx from the EGFL7 promoter. A deletion analysis of the EGFL7 promoter revealed that LANA could activate EGFL7 promoter only when an Ets-1 biding site was present. Irrespective of Daxx overexpression, LANA had no effect on the EGFL7 promoter truncation without the Ets-1 binding site. Additionally, the association of Daxx with Ets-1 and the association of Daxx on the EGFL7 promoter were reduced in the presence of LANA. Collectively, these results suggest that the sequestration of Daxx is the primary mechanism behind the LANA-mediated transactivation of the EGFL7 promoter. Further evidence to support this hypothesis arose after comparing the ability of LANA to transactivate the EGFL7 promoter constructs containing binding sites for all of the transcription factors (-1670 to +100), with a promoter construct containing Ets-1 and GATA binding sites (-150 to +100) and a promoter construct with only GATA binding sites (without the Ets-1 binding site) (-71 to +100). LANA-mediated transactivation of the full-length EGFL7 promoter and the promoter containing Ets/GATA binding sites (-150 to +100) was fairly similar. However, the promoter construct lacking Ets-1 binding sites, but intact GATA binding sites, was unaffected by LANA, suggesting that LANA contributes to the activation of EGFL7 promoter through Ets-1. Schematic of how LANA disrupts the binding of Daxx from Ets-1 to release the repressive effects of Daxx is presented in Figure 9.

While the sequestration of Daxx appears to be the primary mechanism behind the upregulation of the EGFL7 promoter by LANA, the possibility of LANA transactivating the EGFL7 promoter through additional mechanisms still remains. LANA has been previously shown to activate hypoxia inducible factor (HIF)-ɑ [30, 31, 46, 47]. Recently, EGFL7 has been identified as a gene induced in response to HIF-1ɑ. Several studies have suggested that the expression of HIF-1ɑ potentiates the expression of Ets-1, although the underlying mechanism is not clear [48–50]. The possibility of higher EGFL7 expression due to a high level of Ets-1 through HIF-ɑ was also considered but the LANA expressing BJABs and the KSHV infected cells exhibited almost similar levels of Ets-1, confirming the displacement of Daxx from the EGFL7 promoter is the most likely mechanism of LANA mediated upregulation of EGFL7.

The functional significance of EGFL7 upregulation by LANA was demonstrated by comparing the *in vitro* tubulogenic potential of HUVEC cells that did not express LANA, the cells that expressed LANA, and the cells that expressed LANA but had reduced levels of EGFL7 achieved by RNA interference. LANA-expressing cells showed higher tubulogenesis compared with the control cells. However, when the EGFL7 was depleted from these LANA-expressing cells, tubulogenesis was compromised. Although LANA has been demonstrated to induce angiogenesis through multiple mechanisms [8, 29, 30, 42], the knockdown of EGFL7 in LANA-expressing cells compromises tubulogenesis highlights the significant contribution of EGFL7 in LANA-mediated angiogenesis.

In summary, we have identified EGFL7 as an angiogenic cytokine induced during KSHV infection and have explored the mechanism by which a viral latent protein regulates the expression during KSHV infection. EGFL7 controls blood vessel development by promoting endothelial cell proliferation, migration and sprouting during angiogenesis (Figure 9) therefore anti-angiogenic therapy is a promising avenue for the treatment of KS [14, 51]. Targeting EGFL7 in combination with the other currently available anti-angiogenic therapies may increase the efficacy of the currently available anti-angiogenic regimen available for KS treatment. Furthermore, because of the highly-restricted pattern of EGFL7 expression during normal physiological conditions, one can expect anti-EGFL7 therapy to be relatively safer than other anti-angiogenic therapies that target molecules with broader expression profiles and pleiotropic effects. This study lays the foundation for future research focusing on the evaluation of EGFL7 as a potential anti-angiogenic target for the treatment of KS.

## MATERIALS AND METHODS

### Cell culture

The EBV- and KSHV-negative Burkitt lymphoma cell line, BJAB and the KSHV-positive but EBV-negative primary effusion lymphoma cell lines, BC3 and BCBL-1 were cultured in RPMI 1640 medium supplemented with 10% fetal bovine serum, 2mM L-glutamine and penicillin-streptomycin (5 U/ml and 5 ug/ml, respectively). The HUVEC cells were cultured in Medium 200 supplemented (Gibco, USA) with low serum-growth supplement (Gibco), 5 U/ml penicillin and 5 g/ml streptomycin. HUVECs with fewer than 8 passages were used. Human embryonic kidney cells HEK293L were cultured in Dulbecco’s modified Eagle’s medium (DMEM) supplemented with 10% fetal bovine serum, 2mM L-glutamine and penicillin-streptomycin (5 U/ml and 5 g/ml, respectively). All cell lines were grown at 37°C in a humidified chamber supplemented with 5% CO_2_.

### Plasmids

Myc-tagged full-length LANA (pA3M-LANA), or Flag-tagged full-length LANA (pA3F-LANA), lentiviral-constructs, pLVxAcYFP-C1-LANA-Flag and the control vector, pLVxAcYFP-C1-Flag, have been described previously [52]. Flag-Daxx/PRK5 was obtained from Addgene (USA) (Cat#. 27974). Full-length (-1670 to +100) EGFL7 promoter-luciferase reporter construct was purchased from Addgene (Cat# 32244). The truncations mutants of EGFL7 promoter (-150 to +100 and -71 to +100) were further subcloned into PGL3basic vector using an In-Fusion cloning kit (Takara/Clontech, USA) according to the manufacturer’s instructions. The primers sequences used for this cloning were:

(-71 to +100) EGFL7 promoter-luciferase Forward:

5’-AGCTCTTACGCGTGCTAGCATCCCAATCCCGATTACCCA-3’,

(-150 to +100) EGFL7 promoter-luciferase Forward:

5’-AGCTCTTACGCGTGCTAGCCTCAGCCTCCTGTTTGTCCGA-3’,

(+100) EGFL7 promoter-luciferase Reverse:

5’-AGTACCGGAATGCCAAGCTTTGGACCCTAGCCCTTGCTGG-3’.

### Antibodies

The following commercial antibodies were used: Mouse anti-EGFL7 (sc-373898, Santa Cruz, USA), Rabbit anti-EGFL7 antibody (ab115786, Abcam), mouse anti-Daxx (sc-8043, Santa Cruz), rabbit anti-Ets1 (sc-350, Santa Cruz), mouse anti-GAPDH (US Biological, USA), mouse anti-Flag M2 (Sigma Aldrich, USA) and mouse anti-Myc 9E10 (Sigma-Aldrich). Mouse anti-LANA monoclonal antibody was generated at Genscript (USA).

### Immunohistochemistry

Sections of KS biopsy specimen were obtained from the AIDS and Cancer Specimen Resource (ACSR). Tissue sections were subjected to deparaffinization with Histochoice clearing reagent and hydrated with water. The slides were treated with 10mM citrate buffer, pH 3.0, for 30min at 37°C for antigen retrieval then blocked with blocking solution (2% Normal Goat Serum and 0.2% Triton X-100 in PBS). Slides were incubated with primary antibodies, anti-EGFL7 (Abcam Inc.) and anti-LANA (generated at Genscript) separately at 37°C for 1h in a staining chamber followed by treating with HRP-conjugated respective secondary antibodies at 37°C for 1h and developing using DAB reagent (DAKO). The slides were counter-stained with hematoxylin and examined under microscope (Nikon, Inc.) and photographed.

### Lentivirus production

LANA-expressing (YFP-LANA-Flag) and control (YFP-Flag) lentiviral particles were produced using Vesicular stomatitis virus-G envelope-pseudotyped lentiviral virions by co-transfecting 20 μg lentiviral constructs (pLVx-AcYFP-C1-LANA-Flag or pLVx-AcYFP-C1-Flag), along with pseudotyped lentiviral packaging vectors 6.0 μg REV, 10.0 μg GP and 3.0 μg VSV-G into a 10 cm dish of ∼85% confluent HEK293T cells using polyethylenimine as described previously [52]. Eight h post-transfection, the medium was changed and the cells were induced using 10 mM Sodium Butyrate in DMEM supplemented with 10% fetal bovine serum, 2 mM L-glutamine and penicillin-streptomycin (5 U/ml and 5 g/ml, respectively) buffered with 10 mM HEPES (4-(2-hydroxyethyl)-1-piperazineethanesulfonic acid). The media was changed 24 h after transfection to remove the Sodium Butyrate. The supernatant containing the lentiviral particles was collected, passed through a 0.45 μm filter and concentrated to 1/120 of its original volume. The target cells (BJAB) were pre-incubated with polybrene (Hexadimethrine bromide) at the final concentration of 8 μg/ml for 5 minutes before adding the concentrated lentiviral particles. Cells were incubated for 6-8h with the virions followed by changing the medium to remove polybrene. The transduced cells were selected with 2 μg/ml puromycin to obtain a pure population of cells expressing LANA.

### RNA sequencing

The RNA-seq of BJAB cells stably expressing LANA (BJAB-L-YFP) and the control cells (BJAB-YFP) was performed on a HiSeq next-generation sequencer (Illumina, Inc., USA) as described previously [40]. The total RNA was isolated with TRIzol reagent following the manufacturer’s instructions (Life Technologies, USA). The concentration and purity of the extracted RNA was determined using a Nano Drop 2000c spectrophotometer (Nano Drop Technologies, USA). A TrueSeq RNA sample preparation kit v2 (Illumina, Inc.) was used to prepare the cDNA libraries for RNA-seq according to the manufacturer’s instructions. The fragment sizes and purity of the mature libraries were confirmed using a Bioanalyzer 2100 (Agilent Technologies, USA). The quantities of the libraries required for RNA-seq were determined by real-time qPCR using a KAPA library quantification kit for the Illumina platform (Kapa Biosystems, USA). Equal amounts of libraries were sequenced using HiSeq (Illumina, Inc.), and the sequences were mapped to reference human genome (hg19) using the RNA-Seq Analysis tool of CLC Genomic Workbench 9 (CLC Bio, Denmark) software. Relative gene expressions were determined as RPKM (Reads Per Kilobase of transcript per Million mapped reads) using CLC Genomic Workbench 9 (CLC Bio, Denmark). The modulated genes analyzed for pathways using Ingenuity pathways analysis software (Qiagen Inc.)

The RNAseq data from BJAB expressing LANA and control cells are submitted to Gene Expression Omnibus with the accession number GSE82184.

### Chromatin immunoprecipitation assays

Chromatin immunoprecipitation was performed as described previously [52]. Nearly 20 million cells (LANA expressing BJAB and control) were fixed with 1% formaldehyde for 10 minutes at room temperature followed by the addition of glycine at a final concentration of 125mM for 5 minutes to stop cross-linking. The cells were rinsed three times with ice-cold phosphate buffered saline (PBS) and lysed in cell lysis buffer (5mM PIPES (pH 8.0), 85mM KCl and 0.5mM NP-40) supplemented with protease inhibitors for 10 minutes on ice. The nuclei were enriched by low-speed centrifugation and resuspended in protease inhibitor-supplemented nuclear lysis buffer containing 50mM Tris-HCl, pH 8.1, 10 mM EDTA and 1% SDS. Chromatin was sonicated to an average length of 500–800 bp and centrifuged for 10 minutes at 13,000 rpm to remove the cell debris. The resulting supernatant was diluted 5-fold with a ChIP dilution buffer containing 16.7mM Tris-HCl, pH 8.1, 167mM NaCl, 1.2mM EDTA, 0.01% SDS, 1.1% Triton X-100 and protease inhibitors. The diluted chromatin was pre-cleared with Protein A and G sepharose beads pretreated with 1 mg/ml BSA and 1 mg/ml sheared salmon sperm DNA for 30 minutes at 4°C with rotation followed by incubation overnight with either control or specific antibodies at 4°C with rotation. The immune complexes were collected via incubation with Protein A and G sepharose beads for 1–2 h at 4°C. The beads were collected and washed subsequently with a low-salt buffer (0.1% SDS, 1.0% Triton X-100, 2mM EDTA, 20mM Tris [pH 8.1], 150mM NaCl), a high-salt buffer (0.1% SDS, 1.0% Triton X-100, 2mM EDTA, 20mM Tris [pH 8.1], 500mM NaCl), and a LiCl wash buffer (0.25M LiCl, 1.0% NP-40, 1% deoxycholate, 1mM EDTA, 10mM Tris [pH 8.0]). The beads were then washed twice with Tris-EDTA buffer and the chromatin was eluted using an elution buffer (1% SDS, 0.1M NaHCO3) and reverse cross-linked by adding 0.3M NaCl at 65°C overnight. The eluted DNA was precipitated, treated with RNAse and proteinase K at 45°C for 2h and purified using a Min-Elute PCR purification kit (Qiagen, USA). The purified DNA was analyzed by RT-PCR for the presence of the EGFL7 promoter region. Relative binding of Daxx to the EGFL7 promoter was calculated based on the percentage of input samples. Following primer set was for the detection of EGFL7 promoter in a real-time PCR:

Forward: 5’-CCA GCA GGG TCC AGC CCT GGT-3’

Reverse: 5’-TGG GTA ATC GGG ATT GGG ATG-3’

### Low-cell chromatin immunoprecipitation assay:

We performed LowCell ChIP for Daxx from BJAB, BC3, BCBL1 using the LowCell ChIP Kit (Diagenode Inc.). Briefly, 1 million cells were fixed as described above and following a PBS wash cells were re-suspended in the kit-provided chromatin-shearing buffer. Chromatin was sonicated to an average size of 500bp using the Bioruptor Pico (Diagenode Inc.) and chromatin was precipitated as described above.

### Dual luciferase reporter assays

5×10^5^ HEK293L cells or 0.5×10^5^ HUVEC cells were seeded into 6-well plates the day before transfection. 0.5 μg of either wild-type EGFL7 promoter, (-150 to +100) EGFL7 promoter-luciferase, or (-71 to +100) EGFL7 promoter-luciferase plasmids were co-transfected with either variable amounts of Flag-Daxx or pA3F-LANA or both, as indicated in specific experiments. Empty-vector, pA3F was used as a filler plasmid. The transfection efficiencies were monitored by a transfecting green fluorescent protein (GFP) containing vector, pEGFP. Renilla luciferase-expressing plasmid (pRRLSV40) was transfected at 40 ng/well for data normalization. All of the transfections were done using Metafectene (Biontex Laboratories GmbH, Germany) according to the manufacturer’s protocol. Twenty-four h post-transfection the cells were lysed in cell lysis buffer (Promega) and 50 μl of the cell lysate was used for the reporter assay using a dual luciferase reporter assay kit (Promega, USA). The EGFL7 promoter-luciferase readings were normalized against Renilla luciferase to account for the transfection efficiencies and are reported as relative luciferase units. A portion of the cell lysates was used for Western blotting to detect LANA, Daxx and GAPDH. All of the experiments were repeated at least three times and the data shown are the means of three independent experiments.

### Co-immunoprecipitation assays and western blotting analysis

Approximately 10 million cells expressing the proteins of interest were washed with PBS (10mM NaPO_4_, 137mM NaCl, 2.5mM KCl, pH 7.5) and lysed in RIPA cell lysis buffer (50mM Tris-HCl, pH 7.5, 150mM NaCl, 1mM EDTA and 1% NP-40) supplemented with protease inhibitors (1mM phenylmethylsulfonyl fluoride, 10μg/ml pepstatin, 10μg/ml leupeptin and 10μg/ml aprotinin). The cellular lysates were then sonicated to shear the DNA and centrifuged at 12,000 rpm for 10 minutes at 4°C to remove cellular debris. The supernatants were pre-cleared with protein A and G sepharose beads (GE Healthcare, USA) for 30 minutes at 4°C and gently rotated overnight at 4°C with specific antibodies. The resulting immune complexes were captured by the addition of protein A and G conjugated sepharose beads and rotating the lysates for 2 h at 4°C. The immune complexes were collected by centrifuging at 2000 rpm for 2 minutes at 4°C. The beads were washed three times with ice-cold RIPA buffer supplemented with protease inhibitors and boiled in 50 μl of SDS PAGE sample loading buffer for 5 minutes. The cellular lysates and immunoprecipitated proteins were separated on SDS-PAGE gels and transferred onto 0.45 μm nitrocellulose membranes (GE Healthcare, USA) at 100 V for 75 minutes. The blots were then blocked with 5% non-fat milk in TBST buffer (10mM Tris-HCl, pH 7.5, 150mM NaCl, 0.05% Tween 20) and washed three times with TBST buffer before incubating with specific primary antibodies overnight at 4°C. The blots were washed three times with TBST and were then incubated with appropriate secondary antibodies conjugated with Alexa Fluor 680 or Alexa Fluor 800 (Molecular Probes, USA) at 1:10,000 dilutions. The membranes were scanned with an Odyssey scanner (LI-COR, USA).

### EGFL7 ELISA

EGFL7 protein levels in culture supernatant from HUVEC-L-YFP were depleted by immunoprecipitation similarly to the above described methods. Briefly, culture supernatant from HUVEC-L-YFP cells was incubated with control IgG or mouse-anti-EGFL7 (Abnova, Taiwan) at 4°C overnight and EGFL7 protein was collected the following day by the protein A and G conjugated sepharose beads. The resulting supernatants (control-IgG IP’d or EGFL7 depleted-EGFL7 IP’d) were then assessed for the residual levels of EGFL7. To this end, a human EGFL7 protein ELISA specific matched antibody pair (Abnova, Taiwan) was used in sandwich ELISA assay according to the manufacturer’s instructions. ELISA results were read using the EMax Absorbance Microplate Reader (Molecular Devices, USA) with SoftMax Pro v. 6.4 software at an absorbance of 450.

### Quantitative real-time PCR

For quantitative Reverse-Transcriptase PCR assays, we extracted the total mRNA from the cells using an illustra RNAspin Mini kit (GE Healthcare) according to the manufacturer’s instructions. Extracted RNA was subjected to cDNA synthesis using a High-Capacity cDNA reverse transcription kit (Applied Biosystems, USA). Each PCR reaction consisted of 10 μl of 2X SYBR PCR master mix (Applied Biosystems), 1μM of each forward and reverse primers and 2μl of the cDNA. The cDNA was amplified on an ABI StepOne plus real-time PCR machine (Applied Biosystems), and the relative gene copies or the transcripts were calculated with the ΔΔC_T_ method. Each experiment included duplicate samples and the data shown represent the mean of three independent experiments. We calculated P values using two-tailed *t*-tests with Graphpad (Prism 6, USA) software.

### Endothelial tube formation assay

We performed the endothelial tube formation assay using an angiogenesis starter kit (Life Technologies, USA) according to the manufacturer’s instructions. Briefly, the assay was performed using a 24-well micro-titer plate coated with 100 μl of Geltrex Matrix followed by incubating it at 37°C for 30 minutes. The HUVEC cells were re-suspended in Medium 200 and plated on the Geltrex Matrix coated wells at a density of approximately 0.8 × 10^5^ cells per well. The cells were incubated for 24h at 37°C in a humidified chamber before taking the images with a Nikon microscope equipped with camera (Nikon Inc.).

### Statistical analysis

All statistical analyses were performed using Prism 6.0 software (GraphPad Software, Inc., CA, USA) and the statistical significance is indicated on each figure.

## SUPPLEMENTARY MATERIALS TABLE





## References

[1] Cai Q, Verma SC, Lu J, Robertson ES (2010). Molecular biology of Kaposi’s sarcoma-associated herpesvirus and related oncogenesis. Adv Virus Res.

[2] Giffin L, Damania B (2014). KSHV: pathways to tumorigenesis and persistent infection. Adv Virus Res.

[3] Mesri EA, Cavallin LE, Ashlock BM, Leung HJ, Ma Q, Goldschmidt-Clermont PJ (2013). Molecular studies and therapeutic targeting of Kaposi’s sarcoma herpesvirus (KSHV/HHV-8) oncogenesis. Immunol Res.

[4] Dimaio TA, Lagunoff M (2012). KSHV induction of angiogenic and lymphangiogenic phenotypes. Front Microbiol.

[5] Naranatt PP, Krishnan HH, Svojanovsky SR, Bloomer C, Mathur S, Chandran B (2004). Host gene induction and transcriptional reprogramming in Kaposi’s sarcoma-associated herpesvirus (KSHV/HHV-8)-infected endothelial, fibroblast, and B cells: insights into modulation events early during infection. Cancer Res.

[6] Paul AG, Chandran B, Sharma-Walia N (2013). Cyclooxygenase-2-prostaglandin E2-eicosanoid receptor inflammatory axis: a key player in Kaposi’s sarcoma-associated herpes virus associated malignancies. Transl Res.

[7] Prakash O, Tang ZY, Peng X, Coleman R, Gill J, Farr G, Samaniego F (2002). Tumorigenesis and aberrant signaling in transgenic mice expressing the human herpesvirus-8 K1 gene. J Natl Cancer Inst.

[8] Sadagopan S, Sharma-Walia N, Veettil MV, Bottero V, Levine R, Vart RJ, Chandran B (2009). Kaposi’s sarcoma-associated herpesvirus upregulates angiogenin during infection of human dermal microvascular endothelial cells, which induces 45S rRNA synthesis, antiapoptosis, cell proliferation, migration, and angiogenesis. J Virol.

[9] Sharma-Walia N, Paul AG, Bottero V, Sadagopan S, Veettil MV, Kerur N, Chandran B (2010). Kaposi’s sarcoma associated herpes virus (KSHV) induced COX-2: a key factor in latency, inflammation, angiogenesis, cell survival and invasion. PLoS Pathog.

[10] Sivakumar R, Sharma-Walia N, Raghu H, Veettil MV, Sadagopan S, Bottero V, Varga L, Levine R, Chandran B (2008). Kaposi’s sarcoma-associated herpesvirus induces sustained levels of vascular endothelial growth factors A and C early during *in vitro* infection of human microvascular dermal endothelial cells: biological implications. J Virol.

[11] Sodhi A, Montaner S, Patel V, Zohar M, Bais C, Mesri EA, Gutkind JS (2000). The Kaposi’s sarcoma-associated herpes virus G protein-coupled receptor up-regulates vascular endothelial growth factor expression and secretion through mitogen-activated protein kinase and p38 pathways acting on hypoxia-inducible factor 1alpha. Cancer Res.

[12] Ye FC, Blackbourn DJ, Mengel M, Xie JP, Qian LW, Greene W, Yeh IT, Graham D, Gao SJ (2007). Kaposi’s sarcoma-associated herpesvirus promotes angiogenesis by inducing angiopoietin-2 expression via AP-1 and Ets1. J Virol.

[13] Ye FC, Zhou FC, Nithianantham S, Chandran B, Yu XL, Weinberg A, Gao SJ (2013). Kaposi’s sarcoma-associated herpesvirus induces rapid release of angiopoietin-2 from endothelial cells. J Virol.

[14] Nichol D, Stuhlmann H (2012). EGFL7: a unique angiogenic signaling factor in vascular development and disease. Blood.

[15] Luo BH, Xiong F, Wang JP, Li JH, Zhong M, Liu QL, Luo GQ, Yang XJ, Xiao N, Xie B, Xiao H, Liu RJ, Dong CS (2014). Epidermal growth factor-like domain-containing protein 7 (EGFL7) enhances EGF receptor-AKT signaling, epithelial-mesenchymal transition, and metastasis of gastric cancer cells. PLoS One.

[16] Xu HF, Chen L, Liu XD, Zhan YH, Zhang HH, Li Q, Wu B (2014). Targeting EGFL7 expression through RNA interference suppresses renal cell carcinoma growth by inhibiting angiogenesis. Asian Pac J Cancer Prev.

[17] Huang C, Yuan X, Li Z, Tian Z, Zhan X, Zhang J, Li X (2014). VE-statin/Egfl7 siRNA inhibits angiogenesis in malignant glioma *in vitro*. Int J Clin Exp Pathol.

[18] Hansen TF, Christensen R, Andersen RF, Sorensen FB, Johnsson A, Jakobsen A (2013). MicroRNA-126 and epidermal growth factor-like domain 7-an angiogenic couple of importance in metastatic colorectal cancer. Results from the Nordic ACT trial. Br J Cancer.

[19] Huang CH, Li XJ, Zhou YZ, Luo Y, Li C, Yuan XR (2010). Expression and clinical significance of EGFL7 in malignant glioma. J Cancer Res Clin Oncol.

[20] Wu F, Yang LY, Li YF, Ou DP, Chen DP, Fan C (2009). Novel role for epidermal growth factor-like domain 7 in metastasis of human hepatocellular carcinoma. Hepatology.

[21] Diaz R, Silva J, Garcia JM, Lorenzo Y, Garcia V, Pena C, Rodriguez R, Munoz C, Garcia F, Bonilla F, Dominguez G (2008). Deregulated expression of miR-106a predicts survival in human colon cancer patients. Genes Chromosomes Cancer.

[22] Davis DA, Rinderknecht AS, Zoeteweij JP, Aoki Y, Read-Connole EL, Tosato G, Blauvelt A, Yarchoan R (2001). Hypoxia induces lytic replication of Kaposi sarcoma-associated herpesvirus. Blood.

[23] Wang HW, Trotter MW, Lagos D, Bourboulia D, Henderson S, Makinen T, Elliman S, Flanagan AM, Alitalo K, Boshoff C (2004). Kaposi sarcoma herpesvirus-induced cellular reprogramming contributes to the lymphatic endothelial gene expression in Kaposi sarcoma. Nat Genet.

[24] Dupin N, Fisher C, Kellam P, Ariad S, Tulliez M, Franck N, van Marck E, Salmon D, Gorin I, Escande JP, Weiss RA, Alitalo K, Boshoff C (1999). Distribution of human herpesvirus-8 latently infected cells in Kaposi's sarcoma, multicentric Castleman's disease, and primary effusion lymphoma. Proc Natl Acad Sci U S A.

[25] Parravicini C, Chandran B, Corbellino M, Berti E, Paulli M, Moore PS, Chang Y (2000). Differential viral protein expression in Kaposi's sarcoma-associated herpesvirus-infected diseases: Kaposi's sarcoma, primary effusion lymphoma, and multicentric Castleman's disease. Am J Pathol.

[26] Kellam P, Bourboulia D, Dupin N, Shotton C, Fisher C, Talbot S, Boshoff C, Weiss RA (1999). Characterization of monoclonal antibodies raised against the latent nuclear antigen of human herpesvirus 8. J Virol.

[27] Uppal T, Banerjee S, Sun Z, Verma SC, Robertson ES (2014). KSHV LANA--the master regulator of KSHV latency. Viruses.

[28] Paudel N, Sadagopan S, Chakraborty S, Sarek G, Ojala PM, Chandran B (2012). Kaposi's sarcoma-associated herpesvirus latency-associated nuclear antigen interacts with multifunctional angiogenin to utilize its antiapoptotic functions. J Virol.

[29] Watanabe T, Sugaya M, Atkins AM, Aquilino EA, Yang A, Borris DL, Brady J, Blauvelt A (2003). Kaposi's sarcoma-associated herpesvirus latency-associated nuclear antigen prolongs the life span of primary human umbilical vein endothelial cells. J Virol.

[30] Cai QL, Knight JS, Verma SC, Zald P, Robertson ES (2006). EC5S ubiquitin complex is recruited by KSHV latent antigen LANA for degradation of the VHL and p53 tumor suppressors. PLoS Pathog.

[31] Cai Q, Murakami M, Si H, Robertson ES (2007). A potential alpha-helix motif in the amino terminus of LANA encoded by Kaposi's sarcoma-associated herpesvirus is critical for nuclear accumulation of HIF-1alpha in normoxia. J Virol.

[32] Shin YC, Joo CH, Gack MU, Lee HR, Jung JU (2008). Kaposi's sarcoma-associated herpesvirus viral IFN regulatory factor 3 stabilizes hypoxia-inducible factor-1 alpha to induce vascular endothelial growth factor expression. Cancer Res.

[33] Wang X, He Z, Xia T, Li X, Liang D, Lin X, Wen H, Lan K (2014). Latency-associated nuclear antigen of Kaposi sarcoma-associated herpesvirus promotes angiogenesis through targeting notch signaling effector Hey1. Cancer Res.

[34] Mesri EA, Cesarman E, Boshoff C (2010). Kaposi's sarcoma and its associated herpesvirus. Nat Rev Cancer.

[35] Verma SC, Lan K, Robertson E (2007). Structure and function of latency-associated nuclear antigen. Curr Top Microbiol Immunol.

[36] Murakami Y, Yamagoe S, Noguchi K, Takebe Y, Takahashi N, Uehara Y, Fukazawa H (2006). Ets-1-dependent expression of vascular endothelial growth factor receptors is activated by latency-associated nuclear antigen of Kaposi's sarcoma-associated herpesvirus through interaction with Daxx. J Biol Chem.

[37] Wang S, Aurora AB, Johnson BA, Qi X, McAnally J, Hill JA, Richardson JA, Bassel-Duby R, Olson EN (2008). The endothelial-specific microRNA miR-126 governs vascular integrity and angiogenesis. Dev Cell.

[38] Li R, Pei H, Watson DK, Papas TS (2000). EAP1/Daxx interacts with ETS1 and represses transcriptional activation of ETS1 target genes. Oncogene.

[39] Gupta N, Thakker S, Verma SC (2016). KSHV encoded LANA recruits Nucleosome Assembly Protein NAP1L1 for regulating viral DNA replication and transcription. Sci Rep.

[40] Purushothaman P, Thakker S, Verma SC (2015). Transcriptome analysis of Kaposi's sarcoma-associated herpesvirus during de novo primary infection of human B and endothelial cells. J Virol.

[41] Harris TA, Yamakuchi M, Kondo M, Oettgen P, Lowenstein CJ (2010). Ets-1 and Ets-2 regulate the expression of microRNA-126 in endothelial cells. Arterioscler Thromb Vasc Biol.

[42] He M, Zhang W, Bakken T, Schutten M, Toth Z, Jung JU, Gill P, Cannon M, Gao SJ (2012). Cancer angiogenesis induced by Kaposi sarcoma-associated herpesvirus is mediated by EZH2. Cancer Res.

[43] Paudel N, Sadagopan S, Balasubramanian S, Chandran B (2012). Kaposi's sarcoma-associated herpesvirus latency-associated nuclear antigen and angiogenin interact with common host proteins, including annexin A2, which is essential for survival of latently infected cells. J Virol.

[44] Liu YS, Huang ZW, Qin AQ, Huang Y, Giordano F, Lu QH, Jiang WD (2015). The expression of epidermal growth factor-like domain 7 regulated by oxygen tension via hypoxia inducible factor (HIF)-1alpha activity. Postgrad Med.

[45] Ma Q, Cavallin LE, Yan B, Zhu S, Duran EM, Wang H, Hale LP, Dong C, Cesarman E, Mesri EA, Goldschmidt-Clermont PJ (2009). Antitumorigenesis of antioxidants in a transgenic Rac1 model of Kaposi's sarcoma. Proc Natl Acad Sci U S A.

[46] Zhang L, Zhu C, Guo Y, Wei F, Lu J, Qin J, Banerjee S, Wang J, Shang H, Verma SC, Yuan Z, Robertson ES, Cai Q (2014). Inhibition of KAP1 enhances hypoxia-induced Kaposi's sarcoma-associated herpesvirus reactivation through RBP-Jkappa. J Virol.

[47] Cai Q, Lan K, Verma SC, Si H, Lin D, Robertson ES (2006). Kaposi's sarcoma-associated herpesvirus latent protein LANA interacts with HIF-1 alpha to upregulate RTA expression during hypoxia: latency control under low oxygen conditions. J Virol.

[48] Kosaka T, Miyajima A, Shirotake S, Kikuchi E, Hasegawa M, Mikami S, Oya M (2010). Ets-1 and hypoxia inducible factor-1alpha inhibition by angiotensin II type-1 receptor blockade in hormone-refractory prostate cancer. Prostate.

[49] Peters CL, Morris CJ, Mapp PI, Blake DR, Lewis CE, Winrow VR (2004). The transcription factors hypoxia-inducible factor 1alpha and Ets-1 colocalize in the hypoxic synovium of inflamed joints in adjuvant-induced arthritis. Arthritis Rheum.

[50] Tanaka H, Terada Y, Kobayashi T, Okado T, Inoshita S, Kuwahara M, Seth A, Sato Y, Sasaki S (2004). Expression and function of Ets-1 during experimental acute renal failure in rats. J Am Soc Nephrol.

[51] Nikolic I, Plate KH, Schmidt MH (2010). EGFL7 meets miRNA-126: an angiogenesis alliance. J Angiogenes Res.

[52] Thakker S, Purushothaman P, Gupta N, Challa S, Cai Q, Verma SC (2015). KSHV LANA inhibits MHC II expression by disrupting the enhanceosome assembly through binding with the RFX complex. J Virol.

