# Supplementary material for: KSHV LANA upregulates the expression of epidermal growth factor like domain 7 to promote angiogenesis
Source: Oncotarget. 2017 Dec 19;9(1):1210–28. doi: 10.18632/oncotarget.23456 (PMC5787431; doi:10.18632/oncotarget.23456)
Supplement: Supplementary file 1 [file oncotarget-09-1210-s001.pdf]

KSHV LANA upregulates the expression of epidermal growth factor like domain 7 to promote angiogenesis

SUPPLEMENTARY MATERIALS

Supplementary Table 1: List of genes modulated by LANA expression

See Supplementary File 1
